# Supplementary material for: Safety Assessment of Bacteroides Uniformis CECT 7771, a Symbiont of the Gut Microbiota in Infants
Source: Nutrients. 2020 Feb 20;12(2):551. doi: 10.3390/nu12020551 (PMC7071458; doi:10.3390/nu12020551)
Supplement: Supplementary file 1 [file nutrients-12-00551-s001.pdf]

Table S1. Lieberkün crypts depth (µm) and a number of Goblet cells per crypt in all animals (50 animals, n=10 per group), and males and females (25 animals, n = 5 per group), at the end of the safety assessment of *B. uniformis* CECT 7771.

			Control	B_longum10	B_unif8	B_unif9	B_unif10
Lieberkhün crypts	Males	Average	218,49	208,79	211,08	197,66	205,09
		SD	15,49	18,20	9,76	16,60	10,83
	Females	Average	201,98	221,60	203,63	203,89	195,82
		SD	25,38	9,68	15,58	23,98	6,32
	All animals	Average	210,23	215,20	207,35	200,78	200,97
		SD	21,65	15,31	12,87	19,72	9,88
Number of goblet cells/crypt	Males	Average	19,05	20,91	18,03	19,21	17,83
		SD	4,07	3,42	1,59	3,75	3,27
	Females	Average	14,13	18,56	16,77	20,01	15,49
		SD	3,88	3,22	3,83	2,99	2,55
	All animals	Average	16,59	19,74	17,40	19,61	16,79
		SD	4,56	3,37	2,84	3,23	3,05

Samples were assessed from colon sections and measurements were taken in 10 fields for each sample. Average and standard deviation (SD) are shown for all animals (n=10 each group) and males and females (n=5 each group). Statistical significance was considered when a p-value < 0.05.

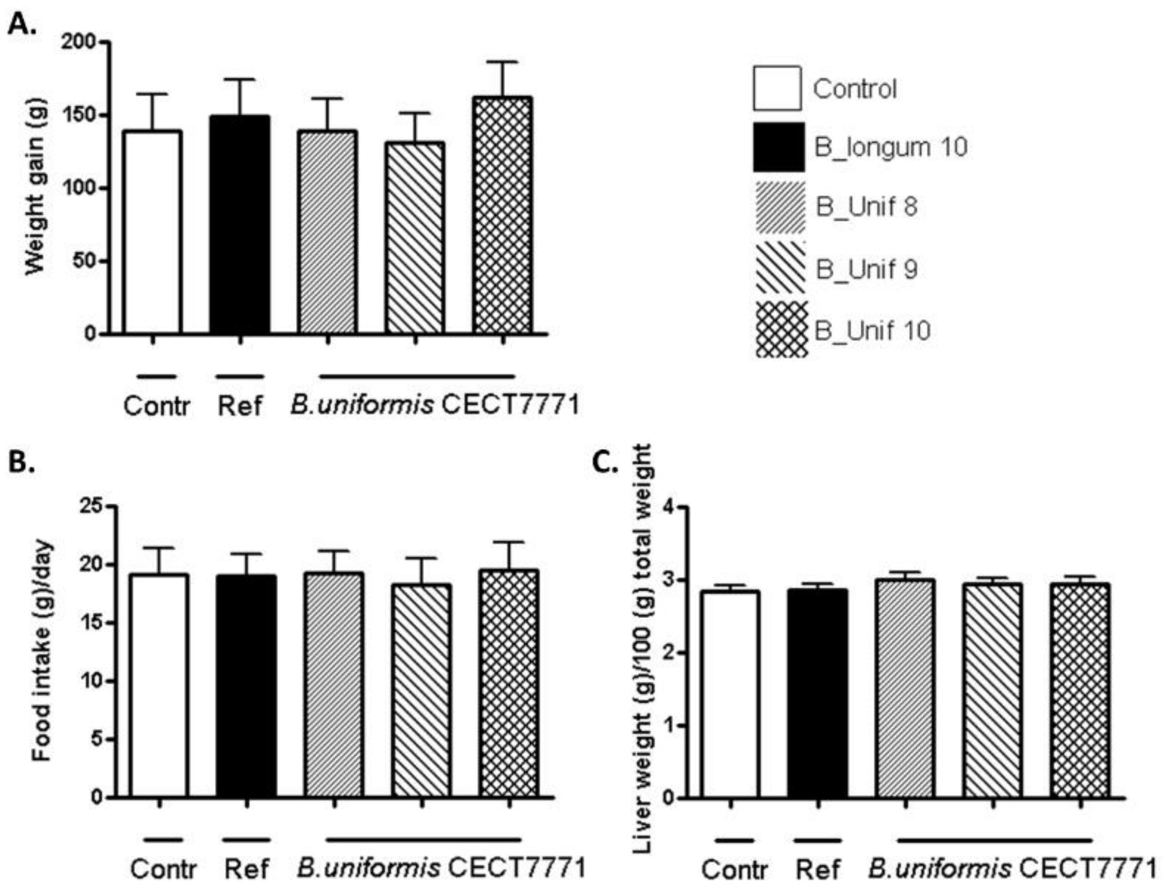

Supplementary figure 1. Body weight gain, food intake and liver tissue weight variation during the safety assessment. **A,** Total body weight gain (g) of all animals during the 90-day assay (50 animals, n = 10 per group). **B,** Daily food intake (g) of all animals during the 90-day assay (50 animals, n = 10 per group). **C**, Liver weight (g) normalised to 100 g of body weight at the end of the study of all animals in the experiment (50 animals, n = 10 per group).

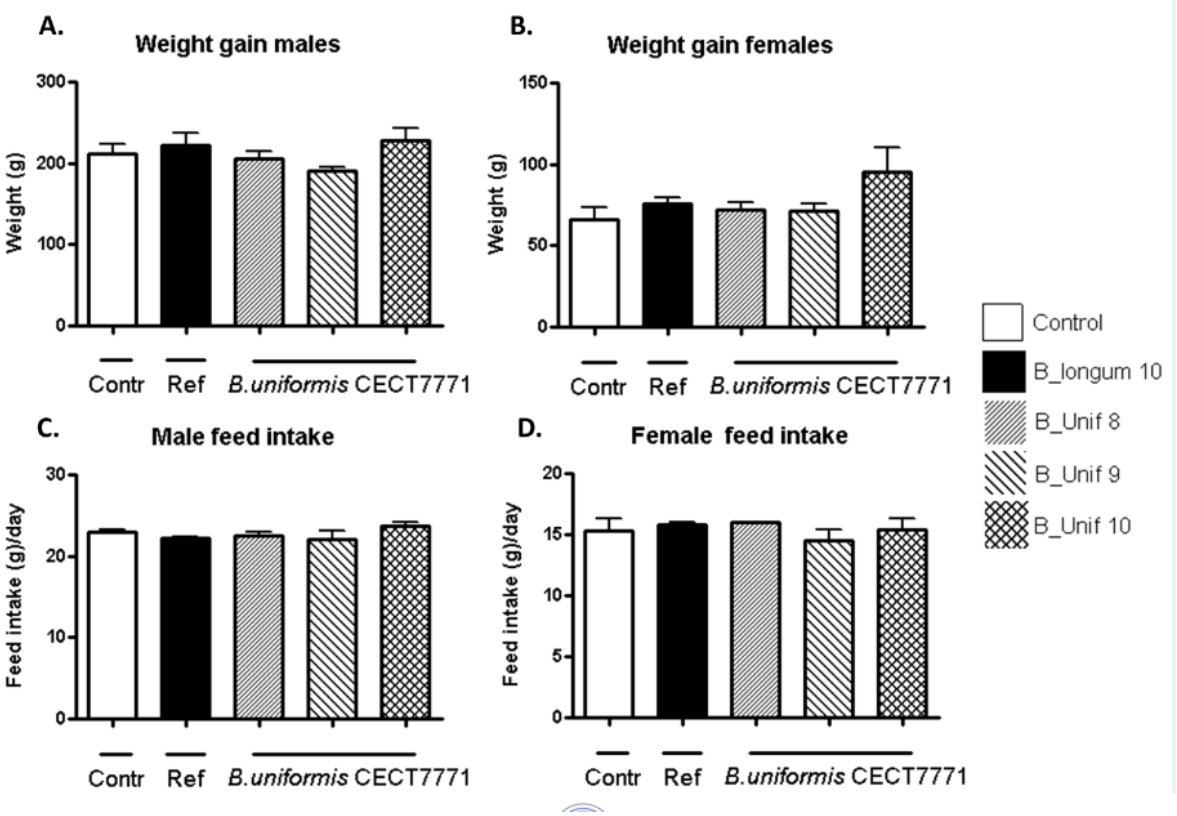

Supplementary figure 2. Body weight gain and food intake during the safety assessment in male and female rats. **A,** Total body weight gain (g) of males during the 90-day assay (25 animals, n = 5 per group); **B,** Total body weight gain (g) of females during the 90-day assay (25 animals, n = 5 per group). **C,** Daily food intake (g) of males during the 90-day assay (25 animals, n = 5 per group). **D**, Daily food intake (g) of females during the 90-day assay (25 animals, n = 5 per group).

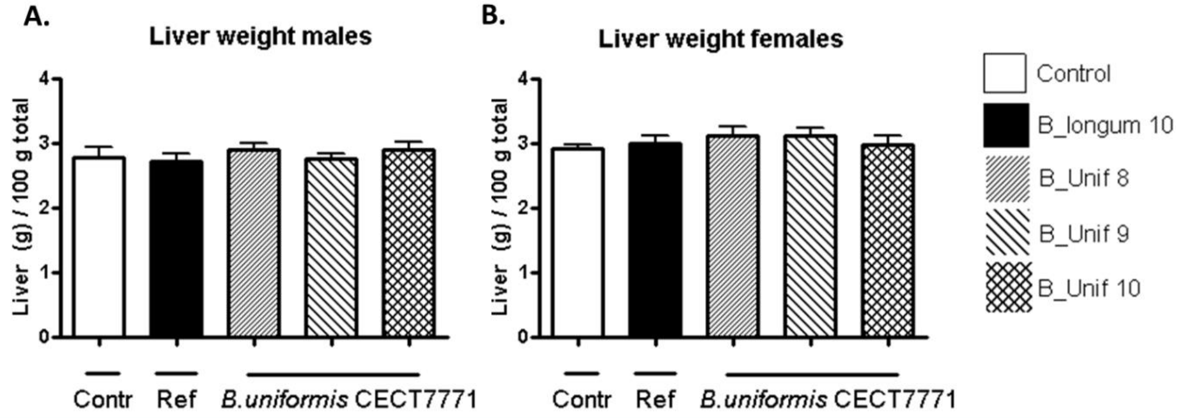

Supplementary figure 3. Liver weight at the end of the study in male and female rats. **A**, Liver weight (g) of males normalised to 100 g of body weight at the end of the study (25 animals, n = 5 per group). **B**, Liver weight (g) of females normalised to 100 g of body weight at the end of the study (25 animals, n = 5 per group).

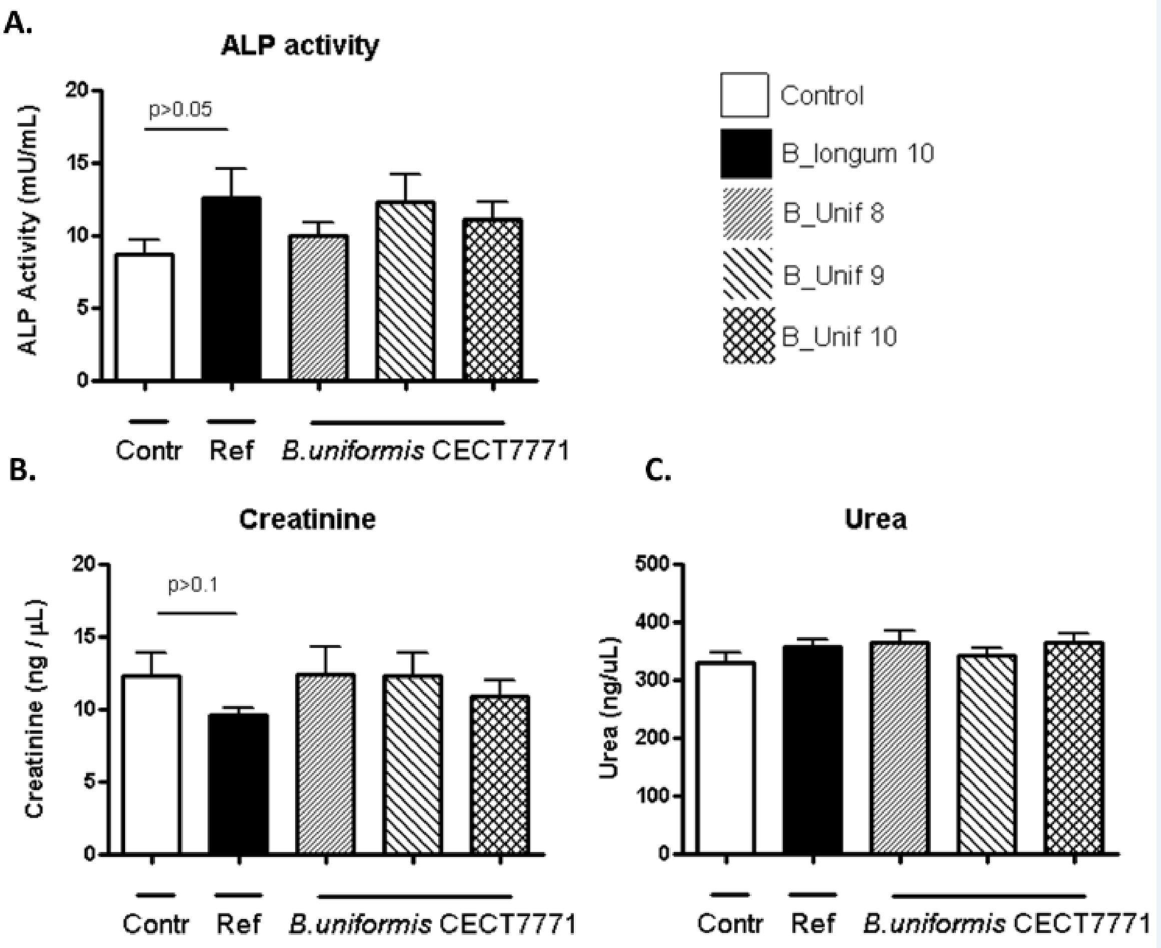

Supplementary figure 4. ALP activity and creatinine and urea concentrations in serum of the experimental groups. **A**, ALP activity (mU/mL) at the end of the study for all groups (50 animals, n = 10 per group). **B**, Creatinine concentration (ng/µL) at the end of the study for all groups (50 animals, n = 10 per group). **C**, Urea concentration (ng/µL) at the end of the study for all groups (50 animals, n = 10 per group). Measurements were made in duplicate. Statistical significance was considered when p < 0.050.

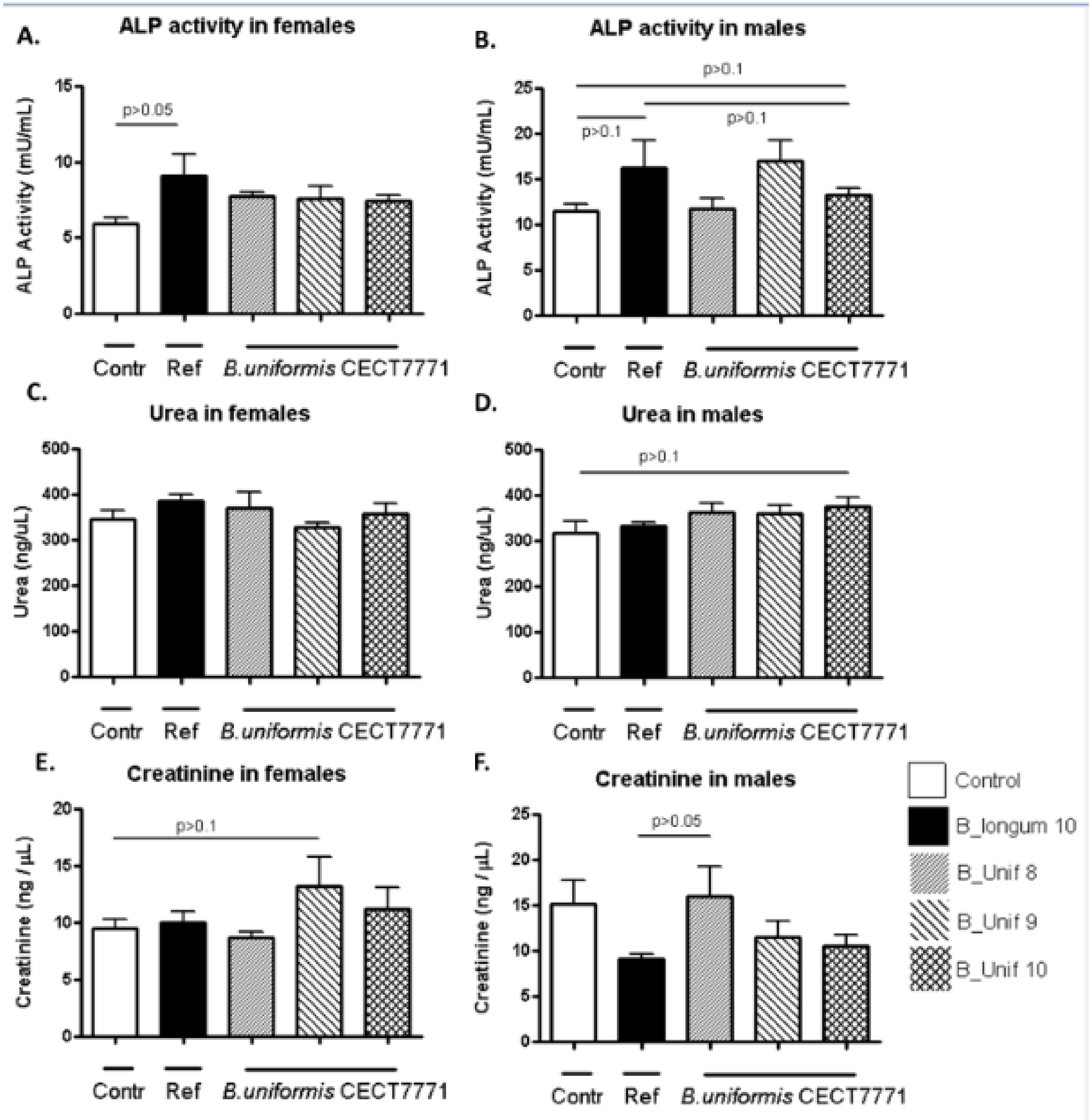

Supplementary figure 5. ALP activity, creatinine and urea concentrations in the serum of male and female rats. **A**, ALP activity (mU/mL) at the end of the study for the females in all groups (25 animals, n = 5 per group). **B**, ALP activity (mU/mL) at the end of the study for the males in all groups (25 animals, n = 5 per group). **C**, Urea concentration (ng/μL) at the end of the study for the females in all groups (25 animals, n = 5 per group). **D**, Urea concentration (ng/μL) at the end of the study for the males in all groups (25 animals, n = 5 per group). **E**, Creatinine concentration (ng/μL) at the end of the study for the females in all groups (25 animals, n = 5 per group). **F**, Creatinine concentration (ng/μL) at the end of the study for the males in all groups (25 animals, n = 5 per group). Measurements were made in duplicate. Statistical significance was considered when $p < 0.050$.

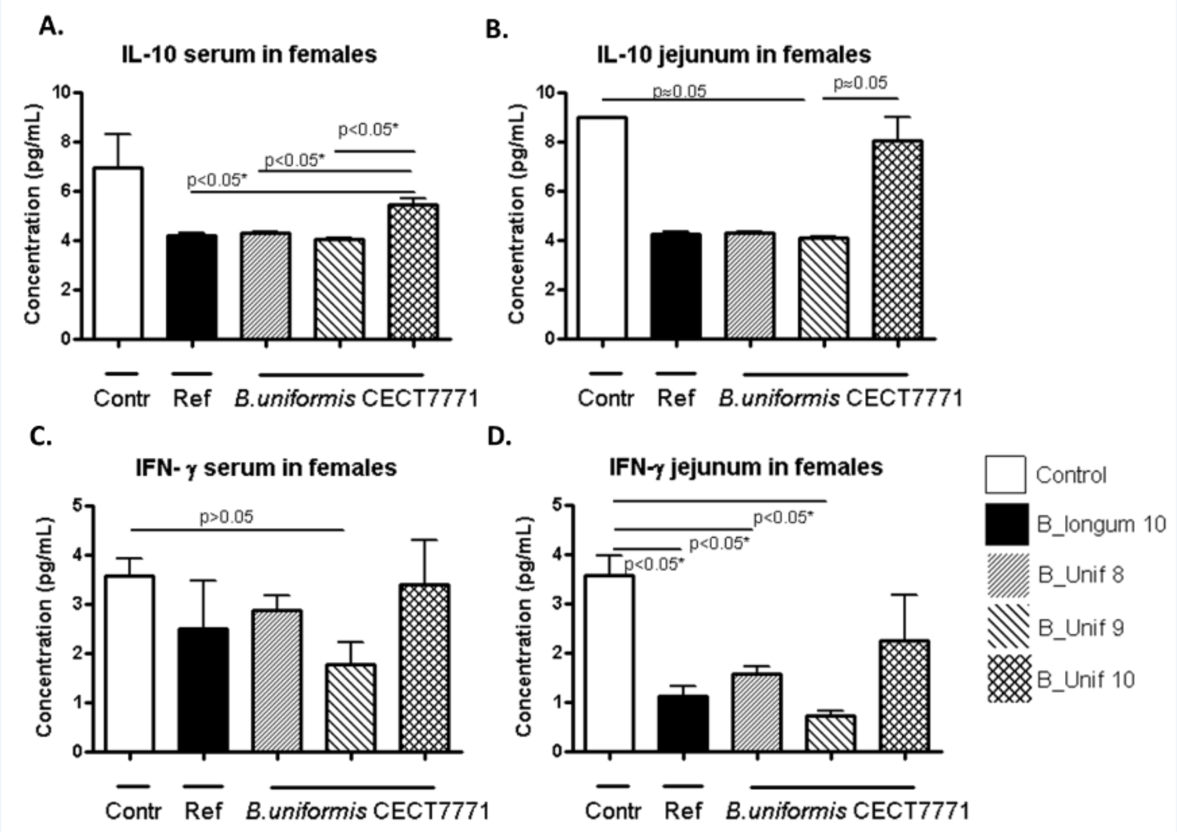

Supplementary figure 6. IL-10 and IFN-γ concentrations in serum and jejunum samples from females. **A**, IL-10 concentration (pg/mL) in the serum at the end of the study for the females in all groups (25 animals, n = 5 per group). **B**, IL-10 concentration (pg/mL) in the jejunum at the end of the study for the females in all groups (25 animals, n = 5 per group). **C**, IFN-γ concentration (pg/mL) in the serum at the end of the study for the females in all groups (25 animals, n = 5 per group). **D**, IFN-γ concentration (pg/mL) in the jejunum at the end of the study for the females in all groups (25 animals, n = 5 per group). Measurements were made in duplicate. Statistical significance was considered when $p < 0.050$.

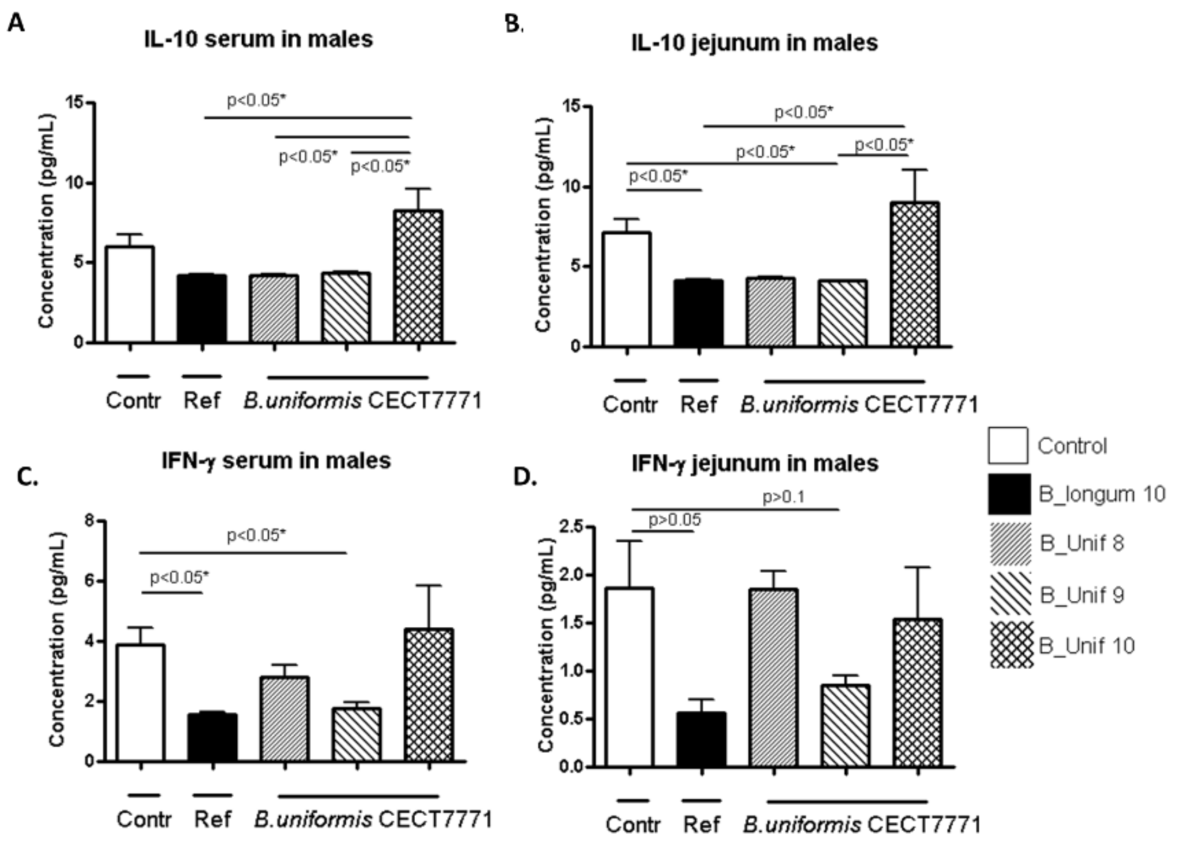

Supplementary figure 7. IL-10 and IFN-γ concentrations in serum and jejunum samples from males. **A**, IL-10 concentration (pg/mL) in the serum at the end of the study for the males in all groups (25 animals, n = 5 per group). **B**, IL-10 concentration (pg/mL) in the jejunum at the end of the study for the males in all groups (25 animals, n = 5 per group). **C**, IFN-γ concentration (pg/mL) in the serum at the end of the study for the males in all groups (25 animals, n = 5 per group). **D**, IFN-γ concentration (pg/mL) in the jejunum at the end of the study for the males in all groups (25 animals, n = 5 per group). Measurements were made in duplicate. Statistical significance was considered when $p < 0.050$.

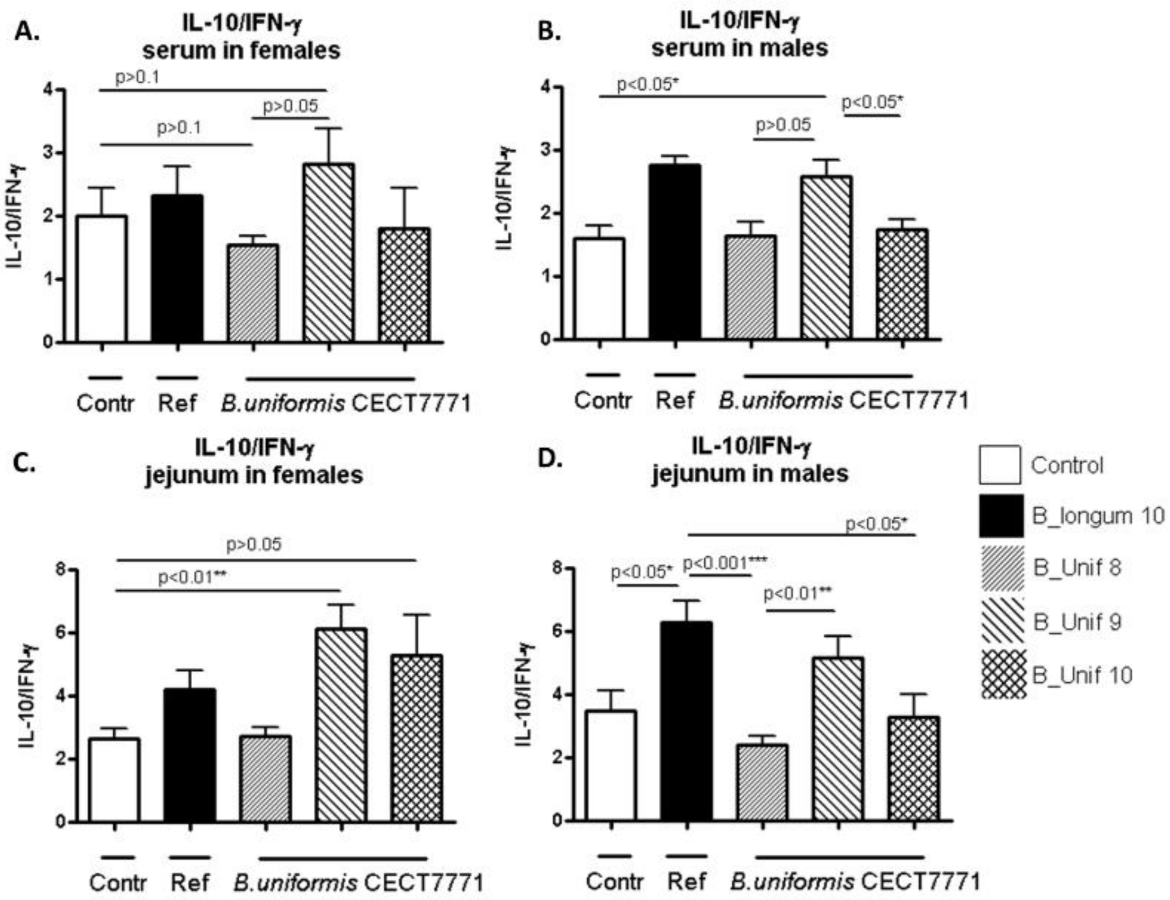

Supplementary figure 8. IL-10/IFN-γ ratios in serum and jejunum samples for males and females. **A**, IL-10/IFN-γ ratios (pg/mL) in the serum samples at the end of the study for the females in all groups (25 animals, n = 5 per group). **B**, IL-10/IFN-γ ratios (pg/mL) in serum samples at the end of the study for the males in all groups (25 animals, n = 5 per group). **C**, IL-10/IFN-γ ratios (pg/mL) in jejunum samples at the end of the study for the females in all groups (25 animals, n = 5 per group). **D**, IL-10/IFN-γ ratios (pg/mL) in jejunum samples at the end of the study for the males in all groups (25 animals, n = 5 per group). Measurements were made in duplicate. Statistical significance was considered when $p < 0.050$.

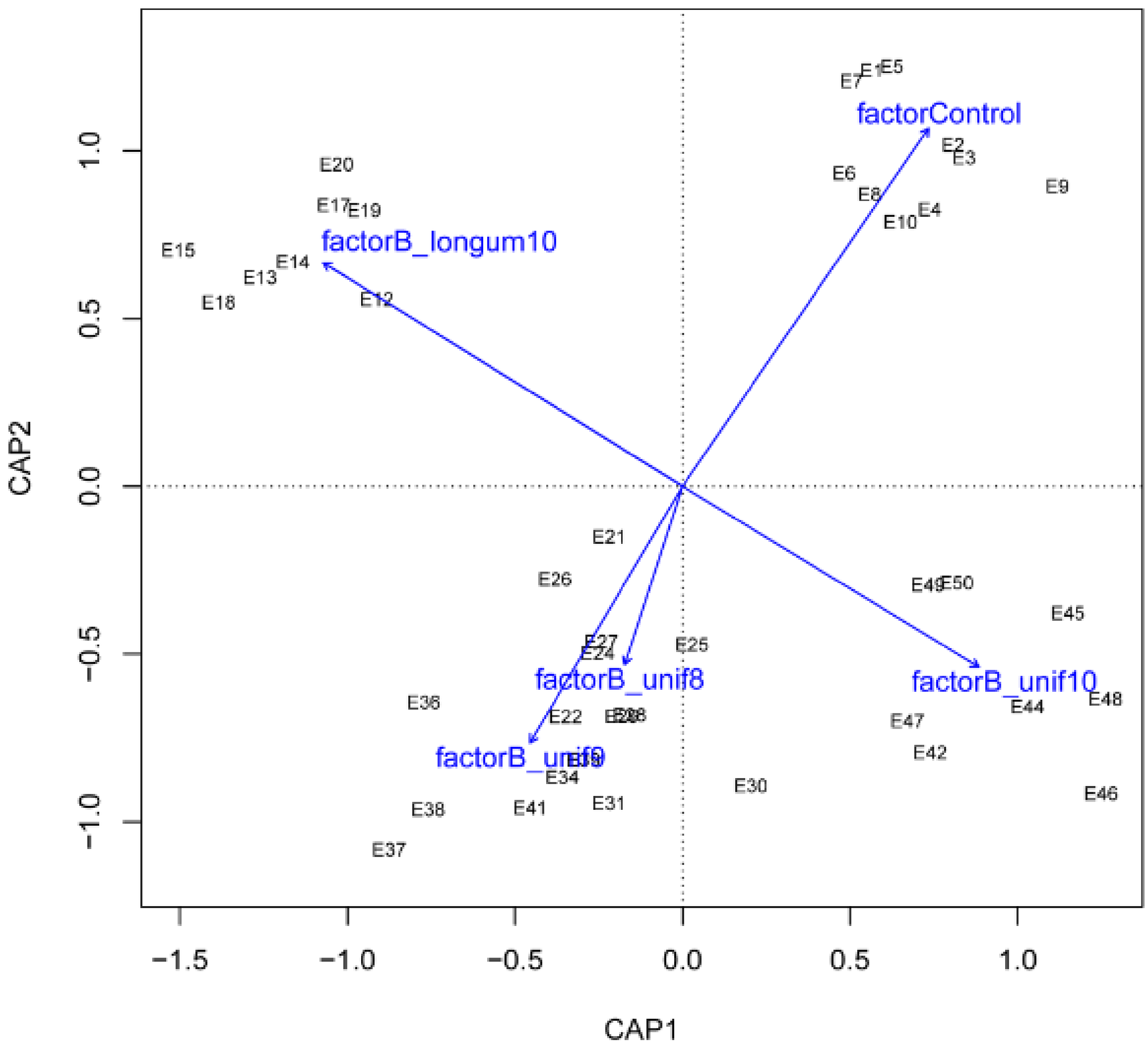

Supplementary figure 9. Distance-based redundancy analysis (dbRDA). The Jaccard-based distance matrix among samples derived from the OTU information was assessed through the RDA interpretative multivariate method to disclose constrained ordination of samples. Vectors (blue lines) and group labels indicate the distribution of samples across multidimensional space according to respective microbiota structures.
